# Traditional Tibetan Medicine in Cancer Therapy by Targeting Apoptosis Pathways

**DOI:** 10.3389/fphar.2020.00976

**Published:** 2020-07-07

**Authors:** Ce Tang, Cheng-Cheng Zhao, Huan Yi, Zang-Jia Geng, Xin-Yue Wu, Yi Zhang, Ya Liu, Gang Fan

**Affiliations:** ^1^ Innovative Institute of Chinese Medicine and Pharmacy, Chengdu University of Traditional Chinese Medicine, Chengdu, China; ^2^ School of Ethnic Medicine, Chengdu University of Traditional Chinese Medicine, Chengdu, China; ^3^ School of Pharmacy, Chengdu University of Traditional Chinese Medicine, Chengdu, China; ^4^ School of Pharmacy, Southwest Minzu University, Chengdu, China; ^5^ Department of Endocrinology, Hospital of Chengdu University of Traditional Chinese Medicine, Chengdu, China

**Keywords:** cancer, traditional Tibetan medicine, anticancer activity, apoptosis, *Ophiocordyceps sinensis*, salidroside, gallic acid

## Abstract

Cancer is a leading cause of death around the world. Apoptosis, one of the pathways of programmed cell death, is a promising target for cancer therapy. Traditional Tibetan medicine (TTM) has been used by Tibetan people for thousands of years, and many TTMs have been proven to be effective in the treatment of cancer. This paper summarized the medicinal plants with anticancer activity in the Tibetan traditional system of medicine by searching for Tibetan medicine monographs and drug standards and reviewing modern research literatures. Forty species were found to be effective in treating cancer. More importantly, some TTMs (*e.g*., *Ophiocordyceps sinensis*, *Phyllanthus emblica* L. and *Rhodiola kirilowii* (Regel) Maxim.) and their active ingredients (*e.g.*, cordycepin, salidroside, and gallic acid) have been reported to possess anticancer activity by targeting some apoptosis pathways in cancer, such as Bcl-2/Bax, caspases, PI3K/Akt, JAK2/STAT3, MAPK, and AMPK. These herbs and natural compounds would be potential drug candidates for the treatment of cancer.

## Introduction

Apoptosis, which is also known as programmed cell death, is beneficial to normal cell development, organ growth, and the dynamic balance of tissues (Rogers and Almenri, 2019). Apoptosis is a normal physiological process that plays an important role in the development and dynamic balance of organisms (Xu et al., 2015). Defects in apoptosis occur in most types of cancer, such as lung, female breast, prostate, liver, thyroid, and bladder cancers. A large number of studies have shown that regulating and inducing apoptosis are feasible ways for treating cancer (Hoshyar and Mollaei, 2017; Yoon et al., 2018). *In vitro* and *in vivo* experiments have demonstrated that the mechanism of apoptosis encompasses extremely complex processes and involve many biological factors, and failure to induce apoptosis is one of the major obstacles to cancer treatment (Li-Weber, 2013). From a mechanistic perspective, apoptosis can be activated by the intrinsic mitochondrial or extrinsic death receptor apoptotic pathway. The intrinsic mitochondrial apoptotic pathway is activated when cells sense directly or indirectly intracellular or extracellular stimuli, such as DNA damage, reactive oxygen species, hypoxia, and Ca^2+^ (Tompkins and Thorburn, 2019). These stimuli ultimately disrupt mitochondrial function by inducing the expression and activation of proapoptotic Bcl-2 family members, such as Bcl-2, Bcl-xL, Bax, and Bak (Hoshyar and Mollaei, 2017). By contrast, stimulated extrinsic death receptors can induce the sequential activation of caspase-3, which cleaves target proteins and leads to apoptosis (Tompkins and Thorburn, 2019). Therefore, the development of anticancer agents with apoptosis pathway-related targets has become an important strategy for cancer treatment.

Natural medicines, including plants, animals, and minerals, are the gifts of nature to humans and play an important role in fighting various diseases. Many anticancer drugs that are commonly used in modern medicine, such as paclitaxel, camptothecin, matrine and vinblastine, are derived directly or indirectly from natural sources. Therefore, new anticancer drugs can be discovered from natural plants. In the course of more than 2,000 years of history, a complete theoretical system has been established for traditional Tibetan medicine (TTM). TTM has played an important role in the prevention and treatment of various diseases, such as “Zhui-nai” (འབྲས་ནད།), which is similar to cancer in modern medicine (Bauer-Wu et al., 2014). TTM believes that “Zhui-nai” is caused by external factors invading the body, resulting in the dysfunction of the three “stomach fire”. These abnormalities can cause indigestion and increase bad blood, which ultimately lead to the dysfunction of the *mei-nian* *loong* (རླུང་མེ་མཉམ།), *neng-xiao* *tripa* (མཁྲིས་པ་འཇུ་བྱེད།), and *baekan* *ni-mu-xie* (བད་ཀན་མྱག་བྱེད།) (Yutuo, 1983). In TTM, unclean substances in the body and physical weakness are important factors in the development of cancer. Therefore, TTM with tonic, heat-clearing and detoxification functions can be used to treat cancer. In recent years, TTM has received extensive attention worldwide owing to its unique advantages in terms of preventing and treating cancer. TTM can directly inhibit the growth of cancer cells, induce apoptosis, and suppress tumor growth through multi-target pathways (Yadav et al., 2017; Bhardwaj et al., 2018; Tao et al., 2019). In addition, TTM combined with radiotherapy or chemotherapy can significantly reduce adverse reactions and enhance the patient’s immunity and quality of life (Liu et al., 2016a; Liu et al., 2016b; Colapietro et al., 2018). Numerous TTM monographs and research papers have documented some natural medicines and prescriptions for cancer treatment. However, no consensus has been reached in most records, resulting in a lack of systematic summarization, induction, and arrangement.

In this study, information on natural Tibetan medicines used in treating cancer was sampled by performing a bibliographic investigation of TTM monographs and drug standards. The names, species, families, and medicinal parts of TTMs with anticancer effect were introduced in detail. These data can provide a good reference for the development and utilization of TTMs. Moreover, recent research progress on some anticancer TTMs and their active ingredients that can induce apoptosis in cancer cells was introduced in detail. These herbs and natural compounds would be potential drug candidates for the treatment of cancer.

## Methods

Some Tibetan medicine monographs and medicinal materials standards, such as “Jing Zhu Materia Medica”, “Drug Standards of Tibetan Medicine” and “Chinese Tibetan Medicine”, were searched for information on natural Tibetan medicine for cancer treatment. Data collected from these documents included names, species, families, and medicinal parts. The botanical names of plants are mainly derived from references, and verified through the “Flora of China (http://frps.eflora.cn/)” and Medicinal Plant Names Services: Royal Botanic Gardens, Kew databases based on their Chinese names. In addition, in order to obtain the active ingredients and biological/pharmacological effects of the selected species, online Chinese databases (*e.g.*, Wanfang and CNKI) and international databases (*e.g.*, NCBI, Web of Science, and Science Direct) were searched with cancer, apoptosis and/or Latin names as search keywords.

## Results and Discussion

### Understanding of Cancer in Traditional Tibetan Medicine

TTM is an important part of traditional medicine worldwide. In the history of more than 2,000 years, TTM has established a complete theoretical system and a unique diagnostic style. It has played an important role in the prevention and treatment of various diseases, including cancer. TTM has a unique understanding of the occurrence and development of cancer. According to the ancient literature of TTM, the hard lump with the size of *Qinggang* nucleus in the body is called “Zhui-nai” (འབྲས་ནད།) (Yutuo, 1983). “Zhui-nai” is similar to cancer in modern medicine. TTM believes that the occurrence of cancer is closely related to “*loong*” and “bad blood”. In general, when the *loong*, *tripa* and *baekan* maintain a relative balance in the body, normal physiological and psychological functions can be achieved. When they are in an unbalanced state, especially the “*loong*” disorder will lead to an increase in “bad blood”, and then the pathological state of “Zhui-nai” is manifested.

**Figure 1:**
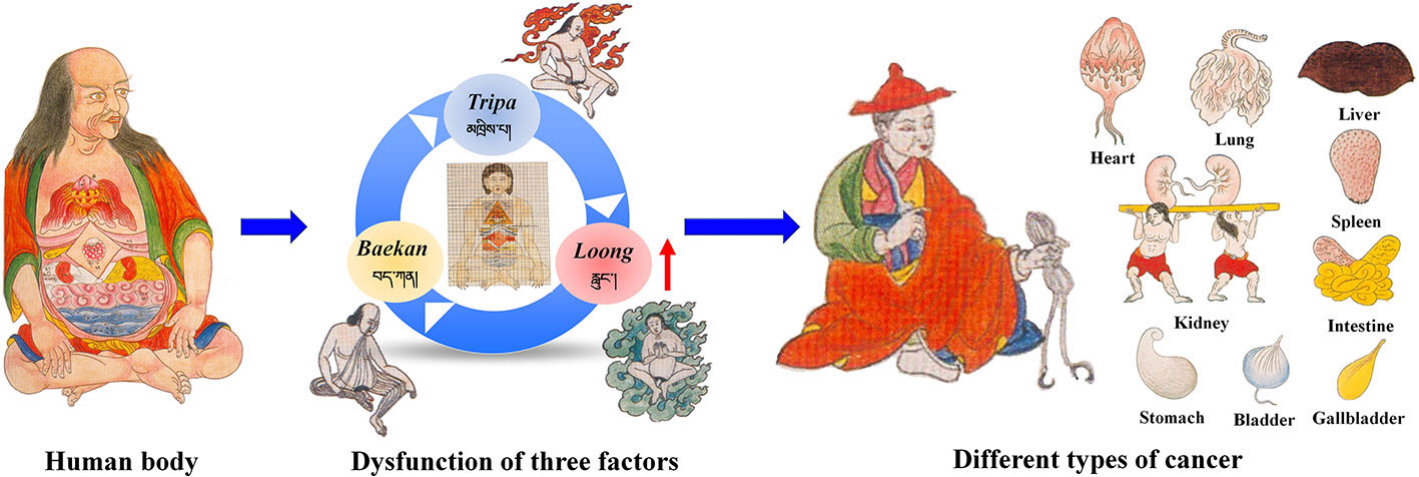
Understanding of cancer in traditional Tibetan medicine system.

The classification of cancer by TTM is generally consistent. According to the “Four Books of Pharmacopeia”, two classification methods, namely, etiology and lesion location classification, are applied (Yutuo, 1983). Eighteen broad types of cancer are classified by etiology classification. By contrast, cancer are classified in to inside and outside according to lesion location classification. Outside cancers can be divided into flesh, bone and pulse cancer, and the inside cancer includes lung, heart, liver, spleen, kidney, stomach, intestine, rectum, and bladder cancers (**Figure 1**). Outside cancers are equivalent to the superficial and soft tissue tumors of modern medicine. Inside cancers mainly refer to abdominal and organ tumors.

**Figure 2:**
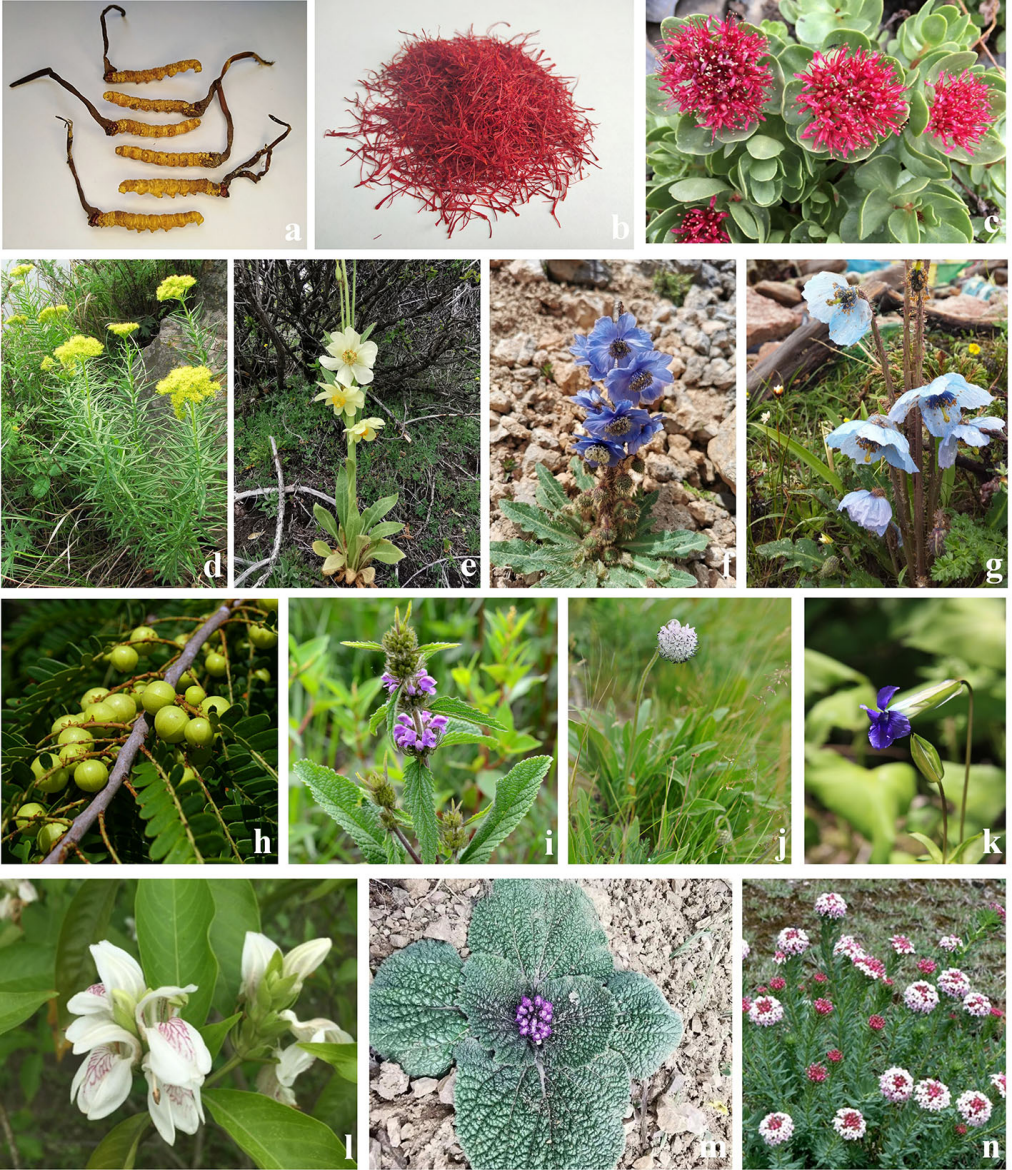
Tibetan medicinal plants with anticancer activity. **(A)** *Ophiocordyceps sinensis* (Berk.) G.H. Sung, J.M. **(B)** Crocus sativus, **(C)** Rhodiola crenulata, **(D)** Rhodiola kirilowii, **(E)** Meconopsis integrifolia, **(F)** Meconopsis racemosa, **(G)** Meconopsis horridula, **(H)** Phyllanthus emblica, **(I)** Phlomis younghusbandii, **(J)** Pterocephalus hookeri, **(K)** Gentianopsis paludosa, **(L)** *Justicia adhatoda* L. (syn. Adhatoda vasica Nees), **(M)** *Phlomoides rotata* (Benth. ex Hook.f.) Mathiesen (syn. *Lamiophlomis rotata* (Benth.) Kudo.), **(N)** Stellera chamaejasme.

The treatment of cancer by TTM can be summarized as follows: The first step is the inhibition, breaking down, and/or dissolution of tumor growth. The second step is the regulation and maintenance of the balance among the *loong*, *tripa*, and *baekan*, cleaning of diseased tissues, and control of inflammation. Finally, target tissues and organs are healed and repaired, and the systemic immune system is restored to normal. In TTM, unclean substances in the body are considered important factors in the development of cancer. Therefore, TTMs with tonic, and heat-clearing, detoxifying functions are usually used to treat cancer (**Table 1** and **Figure 2**). TTM prescriptions for cancer treatment are mainly based on the six tastes (*i.e.*, sweet, sour, salty, bitter, astringent, and pungent). These tastes transform sequentially into three gastropyretic phases, which become into three post-digestive taste profiles (sweet, sour, and bitter) (Bauer-Wu et al., 2014; Dhondrup et al., 2019). Prescription medicines include medicinal plants, animals, and/or minerals, which are processed by powdering, boiling, concentration, and mixing (Dimaer, 2012). TTM also uses some mineral medicines in the treatment of cancer, such as *Margarita*, *Margaritifera concha*, *Magnetitum*, and *Cinnabaris*, but their modern research is scarce.

**Table 1:** Anticancer medicinal plants commonly used in Tibetan traditional medical system.

No.	Latin name	Tibetan name	Family	Used part	Reported anticancer effect		
					Type of extracts	Animal or cell	Effect
1	*Aconitum flavum* Hand. -Mazz.	Bang-na (བོང་ང་ནག་པོ།)	Ranunculaceae	Root	Alkaloid fraction	SGC-7901 HepG2 A549	Inhibition proliferation (Hao, 2014)
					Neolin	SGC-7901 HepG2 A549	Inhibition proliferation (Hao, 2014)
					14-O-acetylneoline	SGC-7901 HepG2 A549	Inhibition proliferation (Hao, 2014)
					Songorine	SGC-7901 HepG2 A549	Inhibition proliferation (Hao, 2014)
					12-epi-napelline	SGC-7901 HepG2 A549	Inhibition proliferation (Hao, 2014)
					12-epi-dehydronapelline	SGC-7901 HepG2 A549	Inhibition proliferation (Hao, 2014)
2	*Amomum tsao-ko* Crevost et Lemaire	Ga-gao-la (ཀ་ཀོ་ལ།)	Zigiberaceae	Fruit	Essential oil	HepG2	Apoptosis induction (Yang et al., 2010)
3	*Anemone rivularis* Buch. -Ham. Ex DC.	Su-ga (སྲུབ་ཀ)	Ranunculaceae	Root and rhizome	Petroleum ether extract	QGY-7703, COLO-205, A549	Inhibition proliferation (Shao et al., 2011)
					Ethyl acetate extract	QGY-7703 COLO-205 A549	Inhibition proliferation (Shao et al., 2011)
					N-butanol part	QGY-7703 COLO-205 A549	Inhibition proliferation (Shao et al., 2011)
4	*Artemisia sieversiana* Ehrh. Ex Willd.	Kan-jia (འཁན་སྐྱ།)	Asteraceae	Aerial part	90% ethanol extract	COLO-205	Apoptosis induction (Tang et al., 2015)
					Achillin	SMMC-7721	DNA damge (Zhang Q. et al., 2004)
					Absinthin	SMMC-7721	DNA damge (Zhang Q. et al., 2004)
5	*Artemisia vestita* Wall. ex DC.	Pu-er-mang-ga-bao (ཕུར་ནག་ལོ་སིབ།)	Asteraceae	Aerial part	Annphenone	HepG2	Inhibition proliferation (Long et al., 2013)
6	*Berberis aristata* D.C	Ji-er-wa (སྐྱེར་པའི་བར་ཤུན།)	Berberidaceae	Stem	Methanolic extract	MCF-7	Apoptosis induction (Serasanambati et al., 2015)
					95% alcohol extract	Mice	Inhibition of tumor growth (Pai et al., 2011)
7	*Carthamus tinctorius* L.	Dun-ge-ri-gong (ལྡུམ་གུར་གུམ།)	Asteraceae	Flower	Dichloromethane extract	Sw620	Apoptosis induction, inhibitory proliferation (Arpornsuwan et al., 2012)
					Hydroxyl safflower yellow A	Mice	Inhibition tumor growth (Ma et al., 2019)
8	*Carum carvi* L.	Guo-niu (གོ་སྙོད།)	Umbelliferae	Fructus	Essential oil	HT-29	Apoptosis induction (Khatamian et al., 2019)
9	*Chrysosplenium nudicaule* Bunge	Ya-ji-ma (གཡའ་ཀྱི་མ།)	Saxifragaceae	Whole plant	6,7,3′-Trimethoxy-3,5,4′-trihydroxy flavone	SGC-7901	Apoptosis induction (Luo et al., 2016)
10	*Crocus sativus* Linn.	Gou-ri-gou-mu (གུར་གུམ།)	Asteraceae	Flower	Crocin	HCT-116 HT-29 SW-480	Apoptosis induction, inhibition proliferation (Aung et al., 2017; Hoshyar and Mollaei, 2017)
11	*Dracocephalum tanguticum* Maxim.	Zhi-yang-gu (པྲི་ཡང་ཀུ།)	Lamiaceae	Aerial part	Chloroform extract	T98G	Inhibition proliferation (Wang et al., 2011)
12	*Entada phaseoloides* (L.) Merr.	Qing-ba-xiao-xia (མཆིན་པ་ཞོ་ཤ)	Leguminosae	Seed	Water soluble extract	K562 U937 HL60	Inhibition proliferation (Xu et al., 2005)
					Total saponins	Mice	Inhibition of tumor growth (Deng et al., 2012)
13	*Gentiana waltonii* Burk.	Jie-ji-na-bao (ཀྱི་ལྕེ་ནག་པོ།)	Gentianaceae	Root	Waltonitone	BEL-7402	Inhibition cell growth (Zhang et al., 2009; Zhang Z.et al., 2010)
14	*Gentianopsis paludosa* (Hook.f.) Ma	Jia-di-na-bu (ལྕགས་ཏིག་ནག་པོ།)	Gentianaceae	Whole plant	95% ethanol extract	SW480	Apoptosis induction (Lu et al., 2016)
					1,7-Dihydroxy-3,8-dimethoxyxanthone	HepG2 HL-60	Inhibition proliferation (Ding et al., 2011)
					1-Hydroxy-3,7,8-trimethoxyxanthon	HepG2 HL-60	Inhibition proliferation (Ding et al., 2011)
15	*Hippophae rhamnoides* L.	Da-bu-kan-za (སྟར་བུ་ཁཎྜ།)	Elaeagnaceae	Fruit	Polysaccharide	Mice	Immunostimulating effect (Wang et al., 2015)
16	*Iris lactea* Pall. var. chinensis Roidz.	Mu-zhe (མོ་གྲེས།)	Iridaceae	Seed	Pallasone A	K562	Apoptosis induction (Zhang F.G. et al., 2010)
17	*Justicia adhatoda* L. (syn. *Adhatoda vasica* Nees)	Ba-xia-ga (བ་ཤ་ཀ)	Acanthaceae	Stem and leave	2-acetyl-benzylamine	MOLM-14 NB-4	Apoptosis induction (Balachandran et al., 2017)
					Vasicine	LLC	Inhibition proliferation (Zhu X.M. et al., 2013)
18	*Lagopsis supina* (Steph) IK.-Gal.	Xing-tuo-li (ཞིམ་ཐིག་ལེ།)	Lamiaceae	Aerial part	95% ethanol extract	HCT-116	Inhibition proliferation (Fang et al., 2018)
19	*Lagotis brevituba* Maxim.	Hong-lian (ཧོང་ལེན།)	Scrophulariaceae	Whole plant	N-butanol extract	Mice SGC-7901	Apoptosis induction, inhibitory tumor growth (Wang, 2006)
20	*Meconopsis horridula* Hook. f. et Thoms.	Ci-er-en (ཚེར་སྔོན།)	Papaveraceae	Whole plant	90% ethanol extract	L1210	Apoptosis induction, inhibition tumor growth (Fan et al., 2015b)
21	*Meconopsis integrifolia* (Maxim.) Franch.	Wu-bai-en-bu (ཨུཏྤལ་སྔོན་པོ།)	Papaveraceae	Whole plant	95% ethanol extract	K562	Apoptosis induction (Fan et al., 2015a)
22	*Meconopsis racemosa* Maxim.	Wu-bai-en-bu (ཨུཏྤལ་སྔོན་པོ།)	Papaveraceae	Whole plant	95% ethanol extract	K562	Apoptosis induction (Fan et al., 2013)
23	*Mirabilis himalaica* (Edgew.) Heim.	Ba-zhu (བ་སྤྲུ།)	Nyctaginaceae	Root	Mirabijalone E	Mice A549	Inhibition proliferation and tumor growth (Linghu et al., 2014)
24	*Ophiocordyceps sinensis* (Berk.) G.H. Sung, J.M.	Ya-er-za-bu-geng (དབྱར་རྩྭ་དགུན་འབུ།)	Clavicipitaceae	Caterpillar body and stroma	Water-soluble polysaccharide	B16-F10	Inhibition migration (Jayakumar et al., 2014)
					Protein extract	A549	Apoptosis induction (Wang Y.X. et al., 2018)
					Cordycepin	B16-F10 Mice	Antimetastatic effect (Nakamura et al., 2015)
25	*Oxytropis flacata* Bunge	E-da-xia (སྔོ་སྟག་ཤ)	Leguminosae	Whole plant	Total alkaloids	Mice	Immunomodulatory effect (Chen et al., 2011)
					Serum containing liposoluble alkaloids	A549	Apoptosis induction (Cheng et al., 2018)
					Total flavonoids	SMMC-7721	Apoptosis induction (Chen et al., 2017)
					Aqueous extract	MCF-7	Apoptosis induction, inhibition proliferation (Zhaxi et al., 2012)
26	*Phlomoides younghusbandii *(Mukerjee) Kamelin & Makhm.	Lu-mu-er (ལུག་མུར།)	Lamiaceae	Root	Phlomiol	Mice K562 Hela	Inhibition proliferation (Xie et al., 2010)
27	*Phlomoides rotata* (Benth. ex Hook.f.) Mathiesen (syn. *Lamiophlomis rotata* (Benth.) Kudo.)	Da-ba (རྟ་ལྤགས།)	Lamiaceae	Aerial part/Whole plant	Essential oil	SGC-7901, BEL-7402, HL-60	Inhibition proliferation (Jia et al., 2005)
					Petroleum ether extract	Tca8113	Apoptosis induction, inhibition proliferation (Kang, 2016; Zheng, 2017)
					Ethanol extract	MEC-1	Apoptosis induction, inhibition proliferation (Ma, 2017)
28	*Phyllanthus emblica* Linn.	Ju-ru-re (སྐྱུ་རུ་ར།)	Euphorbiaceae	Fruit	Tannin fraction	NCI-H1703	Apoptosis induction (Zhao G. et al., 2015)
					Aqueous extract	Mice A549 HepG2 HeLa MDA-MB-231SK-OV3 SW620	Apoptosis induction (Ngamkitidechakul et al., 2010)
					Geraniin	MCF-7	Immunomodulatory effect (Liu X.L. et al., 2012)
29	*Pterocephalus hookeri* (C.B. Clarke) Hoeck	Bang-zi-du-wu (སྤང་རྩི་དོ་བོ།)	Dipsacaceae	Whole plant	N-butanol part	Mice Hep3B	Apoptosis induction, inhibition tumor growth (Guo C. et al., 2015)
					N-butanol part	Hep3B	Apoptosis induction, inhibition tumor growth (Guo C.X. et al., 2015)
					Total saponins	SGC-7901 HepG2 AGS MBA-MD-231	Inhibition proliferation (Lei et al., 2011)
30	*Rhodiola crenulata* (Hook. f. et. Thoms.) H. Ohba	Suo-luo-ma-bu (སྲོ་ལོ་དམར་པོ།)	Crassulaceae	Root and rhizome	Phenolic-enriched extract	MDA-MB-231 V14 Mice	Inhibitory proliferation and tumor growth (Tu et al., 2008)
					Phenolic extract	MDA-MB-231 TER-sisSFRP1	Antimetastatic effect (Gauger et al., 2010)
					Phenolic extract	MCF-7	Inhibitory proliferation and tumor growth (Bassa et al., 2016)
					Root extract	U87	Inhibitory proliferation (Mora et al., 2015)
					95% ethanol extract	Mice	Apoptosis induction, inhibitory proliferation and tumor growth (Zhang et al., 2013)
					Salidroside	MDA-MB-231	Apoptosis induction (Ge et al., 2019)
31	*Rhodiola kirilowii* (Regel) Maxim	Bang-shen-ba (སྤང་ཚན་པ།)	Crassulaceae	Root and rhizome	95% ethanol extract	MDA-MB-231	Antimigration effect (Wang and Lin, 2015)
32	*Rhodiola tangutica* (Maxim.) S.H. Fu (syn. *Rhodiola algida* var. tangutica)	Suo-luo-ma-bu (སྲོ་ལོ་དམར་པོ།)	Crassulaceae	Root and rhizome	75% alcohol extract	MCF-7	Apoptosis induction (Lu D. et al., 2011)
					Aqueous extract	MCF-7	Apoptosis induction, inhibitory proliferation (Lu D.X. et al., 2011; Qi et al., 2015)
33	*Sapindus mukorossi* Gaertn.	Long-dong (ལུང་ཏང་།)	Sapindaceae	Seed	Ethyl acetate extract	A375.S2 MeWo A549	Inhibitory proliferation (Chen C.Y. et al., 2010)
					Hexane extract	A375.S2 MeWo A549	Inhibitory proliferation (Chen C.Y. et al., 2010)
34	*Saussurea laniceps* Hand. -Mazz.	Qia-guo-su-ba (བྱ་རྒོད་སུག་པ།)	Asteraceae	Whole plant	Umbelliferone	HepG2	Apoptosis induction (Chen et al., 2016)
35	*Stellera chamaejasme* L.	Re-jia-ba (རེ་ལྕག་པ།)	Thymelaeaceae	Root	Water extract	Mice NCI-H520	Apoptosis induction, inhibition tumor growth (Xing et al., 2009; Yang et al., 2017)
					Alkane extract	Mice	Inhibition of tumor growth (Zhao et al., 2018)
					Aotal alkaloids	SGC-7901 BEL-7402 HL-60	Apoptosis induction (Wang et al., 2010)
36	*Swertia chirayita* (Roxb. ex Flem.) Karst.	Di-da (ཏིག་ཏ།)	Gentianaceae	Whole plant	Methanol extract	Shrimp	Inhibitory tumor growth (Khan et al., 2017)
37	*Swertia mussotii* Franch	Di-da (ཏིག་ཏ།)	Gentianaceae	Whole plant	N-butanol part	MGC-803	Inhibition proliferation (Wang H.X. et al., 2016)
					70% ethanol fraction	Mice MGC-803	Apoptosis induction, inhibition tumor growth (Wang H. et al., 2018)
					100% ethanol fraction	BGC-823	Apoptosis induction (Wang H. et al., 2018)
					4, 6, 8-trihydroxy-1,2, 3,5-tetramethoxyanthone	C6	Inhibition of cell growth (Shi et al., 2018)
38	*Syzygium cumini* (L.) Skeels	Sa-zhe (སྲ་འབྲས།)	Myrtaceae	Fructus	70% ethanol extract	AML	Inhibition proliferation (Afify et al., 2011)
					*γ*-sitosterol	AML	Inhibition proliferation (Afify et al., 2011)
					kaempferol 7-O-methylether	AML	Inhibition proliferation (Afify et al., 2011)
39	*Terminalia chebula* Retz.	A-ru-la (ཨ་རུ་ར།)	Combretaceae	Fruit	70% methanol extract	MCF-7 S115 HOS-1 PC-3 PNT1A	Inhibition proliferation (Saleem et al., 2002)
					Chebulinic acid	COLO-205	Apoptosis induction (Reddy et al., 2009)
					Chebulinic acid	MCF-7 S115 HOS-1 PC-3 PNT1A	Inhibition proliferation (Saleem et al., 2002)
					Ellagic acid	MCF-7 S115 HOS-1 PC-3 PNT1A	Inhibition proliferation (Saleem et al., 2002)
40	*Tinospora cordifolia* (Willd.) Hook.f. & Thomson	Le-zhe (སླེ་ཏྲེས།)	Menispermaceae	Stem	50% ethanol extract	C6	Apoptosis induction, inhibition proliferation (Mishra and Kaur, 2013)
					Alkaloid Palmatine extract	Mice	Inhibition of tumor growth (Ali and Dixit, 2013)

### Apoptotic Pathway as a Target of TTM in the Treatment of Cancer

Apoptosis, which is known as programmed cell death, is a widely important mechanism of cell growth inhibition in cancer cells. Therefore, the apoptotic pathway is an important target for cancer treatment (Tompkins and Thorburn, 2019). By collating the literature of Tibetan medicines with anticancer activity, up to now, these anticancer TTMs were found among forty species, such as *Ophiocordyceps sinensis* (Berk.) G.H. Sung, J.M., *Crocus sativus* L., *Phyllanthus emblica* L., *Rhodiola* species, *Mirabilis himalaica* (Edgew.) Heimerl, *Terminalia chebula* Retz. Some TTMs can kill cancer cells by inducing apoptosis. In the following sections, we will focus on introducing the TTMs and their compounds whose therapeutic mechanisms are related to apoptosis (**Tables 2** and **3**).

**Table 2:** Antiapoptosis mechanism of TTM extract related to cancer.

No.	Cancer type	Extract	Object	Dose	Mechanism	References
1	Glioblastomas	Dracocephalum tanguticum Maxim. (Total saponins)	T98G	90 μg/ml	Bax↑, p21↓, Caspase-3↑	Wang et al., 2011
2	Glioblastomas	*Rhodiola crenulata* (Root extract)	U87	200 μg/ml	β-catenin↓, Wnt↓	Mora et al., 2015
3	Glioblastomas	Tinospora cordifolia (50% ethanolic extract)	C6	250 and 350 μg/ml	Bcl-xL↓, CyclinD1↓, MMP-2↓, MMP-9↓	Mishra and Kaur, 2013
4	Liver cancer	*Pterocephalus hookeri* (n-butanol extract)	Hep3B	20, 50, 100, and 200 μg/ml	Bcl-2↓, Bax↑, Bax/Bcl-2↑, p-Akt↓, p-PDK1↓	Guo C. et al., 2015
5	Liver cancer	*Oxytropis Falcata* (Total Flavonoids)	SMMC-7721	50, 75, and 100 μg/ml	Caspase-3↑, Cyto-c↑	Chen et al., 2017
6	Leukemia	*Meconopsis integrifolia* (95% ethanol extract)	K562	30, 60, and 90 μg/ml	ROS↑, cleaved Caspase-3/9↑, cleaved PARP↑	Fan et al., 2015a
7	Leukemia	*Meconopsis Horridula* Hook (95% ethanol extract)	L1210	60, 90, and 120 μg/ml	ROS↑	Fan et al., 2015b
8	Gastric cancer	*Swertia mussotii* (50% and 100% ethanol extract)	MGC-803 BGC-823	300, 600, and 900 μg/ml	ROS↑, Ca^2+^↑, MMP ↑	Wang H. et al., 2018
9	Cervical cancer	*Phyllanthus emblica* (Polyphenol extract)	HeLa	150 mg/ml	Fas↑, FasL↑, cleaved Caspase-8↑	Zhu X. et al., 2013
10	Colon cancer	Gentianopsis paludosa (Hook. f.) Ma. (95% ethanol extract)	SW480	2, 10, 50, and 250 μg/ml	NF-κB↓	Lu et al., 2016
11	Lung cancer	*Phyllanthus emblica* (Tannin fraction)	NCI-H1703	15, 30, and 60 mg/l	p-ERK/ERK↓, MMP2/9↓, p-JNK/JNK↑	Zhao H.J. et al., 2015
12	Breast cancer	*Rhodiola* *kirilowii* (95% ethanol extract)	MDA-MB-231	10, 20, and 40 mg/ml	p-Akt↓, p-PKC↓	Wang and Lin, 2015

↑: upgrade; ↓: downgrade.

**Table 3:** Antiapoptosis mechanism of the active ingredient of TTM related to cancer.

No.	Cancer type	Active ingredient	Object	Dose	Mechanism	References
1	Lung cancer	Berberine	A549	30, 60, and 90 μM	Bax↑, Bcl-2↓, Bax/Bcl-2↑, JAK2↓, VEGF↓, NF-κB p65↓, AP-1↓, MMP-2↓	Li J. et al., 2018
2	Lung cancer	Berberine	A549 PC9	40, 80, and 120 μM	miR-19a↓, p-JNK↑, p-p38MAPK↑, Bax↑, Bcl-2↓, Bax/Bcl-2↑, TF↓,	Chen et al., 2019
3	Lung cancer	Berberine	A549	6.25, 12.5, 25, 50, and 100 μM	p53↑, FPXP3a↑, p21↑, CyclinD1↓, p-ERK↑, p-p38MAPK↑	Zheng et al., 2014
4	Lung cancer	Gallic acid	H446	3 μg/ml	Bax↑, P53↑, DIABLO↑, APAF-1↑, XIAP↓, ROS↑	Wang R. et al., 2016
5	Lung cancer	Gallic acid	A549	25, and 50 μM	IκBα↑, p-NF-κB p65↓	Choi et al., 2009
6	Lung cancer	Cordycepin	H1975	5.11, 10.22, and 15.34 μM	Bcl-2↓, Bax↑, Caspase-3↓, Cleaved Caspase-3↑, p-EGFR↓, P-Akt↓, p-ERK1/2↓	Wang Z. et al., 2016
7	Lung cancer	Cordycepin	A549	75, 110, and 145 μM	NF-κB p65↓, Bax↑, Bcl-2↓, cleaved Caspase-3↑	Zhang et al., 2015
8	Lung cancer	Mirabijalone E	A549	20 and 40 μg/ml	Bcl-2↓, Bax↑, Bax/Bcl-2↑, Caspase-3↑	Linghu et al., 2014
9	Breast cancer	Salidroside	MDA-MB-231 MCF-7	2.5, 5, and 10 μM	Cleaved-Caspase9↑, Bcl-2↓, Bax↑, Bax/Bcl-2↑	Hu et al., 2010
10	Breast cancer	Salidroside	MDA-MB-231	10, 20, and 40 μM	p-EGFR↓, p-JAK2↓, p-STAT3↓, MMP2/3/9↓, p-STAT5↓, VEGF↓, STAT3↓	Kang et al., 2018
11	Breast cancer	Salidroside	MCF-7	5, 20, and 40 μM	Bcl-2↓, Bax↑, Bax/Bcl-2↑, P21↑, CyclinD3↓, CyclinD1↓, MMP-9↓, MMP-2↓, ROS↓, p-p38MAPK↓, p-ERK1/2↓, p-JNK↓	Zhao G. et al., 2015
12	Breast cancer	Berberine	MDA-MB-231	5, 10, and 20 μg/ml	Caspase-3/9↑, Clv-C3↑, Bax↑, Bcl-2↓, Bax/Bcl-2↑, Lig4↑, Cyto-c↑	Zhao et al., 2017
13	Breast cancer	Berberine	MCF-7	10 and 80 μM	AMPK↑, HIF-1α↓, P-gp↓, p53↑, Bax, Cyto-c↑, cleaved Caspase-3/9↑, cleaved PARP↑	Pan et al., 2017
14	Breast cancer	Berberine	MDA-MB-231 MCF-7	50 μM	ROS↑, AIF↑, JNK↑, Cyto-c↑, Bcl-2↓, Bax↑, Bax/Bcl-2↑, Caspase-3↑,	Subramanian et al., 2016
15	Breast cancer	Crocin	MCF-7	10, 25, and 50 μM	p53↑, Bax↑, Bcl-2↓, Bax/Bcl-2↑, MMP↓, Cyto-c↑	Lu et al., 2015
16	Liver cancer	Berberine	Huh7	5, 10, and 20 μM	PARP↓, cleaved PARP↑, PCNA↓, Bid↓, Bcl-2↓, Pro-Caspase-3/7/9↓	Yip and Ho, 2013
17	Liver cancer	Berberine	HepG2	12.5 and 50 μM	Caspase-3/9↑, Cyto-c↑, Bax↑, Bcl-2↓, p-AMPK/AMPK↑, p-Akt/Akt↓, Bax/Bcl-2↑, p-Akt↑, NF-κB p65↓,	Yang and Huang, 2013; Li et al., 2017
18	Liver cancer	Waltonitone	BEL-7402	25 μM	p-ERK1/2↑, p-Akt↑, p53↑	Zhang et al., 2009 and Zhang Z. et al., 2010;
19	Liver cancer	Cordycepin	HepG2	124, 250, and 500 μM	Bax↑, Bid↓, Fas↑, FADD↑, Pro- Caspase-3/8/9↓, t-Bid↑, Cyto-c↑, Cleaved-Caspase-3/8/9↑	Shao et al., 2016
20	Liver cancer	Ellagic acid	HepG2	1 mM	ROS↑, Bax↑, Bcl-2↓, Bax/Bcl-2↑, p53↑, p21↑, MMP-9↓,	Das et al., 2017
21	Colon cancer	TC-2	HCT-116	7.5, 15, and 30 μM	ROS↑	Sharma et al., 2018
22	Colon cancer	Ellagic acid	HCT-15	60 μM	ROS↑, PCNA↓, Cyclin D1↓, PI3K↓, p-Akt↓, Bax↑, Bcl-2↓, Cyto c↑, Capase-3↑	Umesalma et al., 2015
23	Colon cancer	Chebulagic acid	COLO-205	25 μM	Bcl-2↓, Bax↑, Bax/Bcl-2↑, Cyto-c↑, PARP cleavage	Reddy et al., 2009
24	Colon cancer	Salidroside	SW1116	10, 20, and 50 μg/ml	p-JAK2↓, p-STAT3↓, VEGFR2↓, VEGF↓, MMP-2/9↓	Sun et al., 2015
25	Prostate cancer	Berberine	PC-3	25, 50, 75, and 100 μM	ROS↑, Cyto-c↑, Smac/DIABLO↑, Caspase-9↓, cleaved Caspase-9/3↑, PARP↑	Meeran et al., 2008
26	Prostate cancer	Ellagic acid	PC3	30, 50, and 70 μM	p-STAT3↓, p-Akt↓, p-ERK1/2↓	Eskandari et al., 2016
27	Prostate cancer	Palmatine	DU145	5 and 10 μg/ml	IGF-IR↓, rpS6↓, c-Abl↓, NF-κB Reporter↓, FLIP Reporter↓	Hambright et al., 2015
28	Oral cancer	Berberine	SCC-4	75 μM	ROS↑, Ca^2+^↑, Caspase-3/8/9↑, Bcl-2↓, Bcl-xL↓, Bax↑, Bad↑, Bak↑, Cyto-c↑, APAF-1↑, FADD↑, Fas↑	Ho et al., 2009
29	Oral cancer	Berberine	KB	0.1 and 1 μg/ml	FasL↑, cleaved Caspase-3/8/9↑, Bcl-2↓, Bcl-xL↓, Bax↑, Bad↑, cleaved PARP↑, p-p38MAPK/p38MAPK↑, MMP-2/9↓, p-ERK/ERK↑, Apaf↑	Kim et al., 2015
30	Oral Cancer	Ellagic acid	DMBA-induced HBP carcinogenesis model	0.1, 0.2, and 0.4% in diet	GSK-3β↓, β-catenin↓, NF-κB (p50 and p65) ↓, Bax↑, Bcl-2↓, p-IκB↓, IKKβ↓, IκB↑, cleaved Capase-3↑, PARP↑	Anitha et al., 2013
31	Colorectal cancer	Salidroside	HCT-116	0.5, 1, and 2 μg/ml	LC3B↑, p-AMPK↑, p-NF-κB p65↓, TGFβ1↓, p-STAT3↓, p-mTOR↓, p-JAK2↓,	Li and Chen, 2017
32	Colorectal cancer	Salidroside	HT29	0.5, 1, and 2 mM	Bax/Bcl-2↑, LC3-II/LC-1↑, Beclin-1↑, p-PI3K↓, p-Akt↓, p-mTOR↓	Fan et al., 2016
33	Ovarian cancer	Salidroside	SKOV3 A2780	1,000 μM	Bax/Bcl-2↑, AIF↑, Bad↑, p-Bad↓, p53↑, p31↑, p16↑, XIAP↓, Caspase-3↑	Yu et al., 2018
34	Ovarian cancer	Crocin	HO-8910	0.2, 0.4, 0.8, and 1.0 mM	Fas↑, p53↑, cleaved Caspase-3↑	Xia, 2015
35	Leukemia	2-acetyl-benzylamine	MOLM-14 NB-4	0.42, and 0.84 mM	Bcl-2↓, Bax↑, Cyto-c↑, Caspase-3↑, JAK-2/p-JAK-2↓, STAT-3/p-STAT-3↓, Bax/Bcl-2↑,	Balachandran et al., 2017
36	Leukemia	Salidroside	THP-1 U937	2 mM	LC3II/LC3I↓, Bax/Bcl-2↑, Beclin1↓, p-Akt/Akt↓,Mp-PI3K/PI3K↓, p62↓, p-mTOR/mTOR↓, AMPKα1↑,	Ge et al., 2019
37	Kidney cancer	Salidroside	A498 786-O	15, 30, and 60 μM	Cyclinn B1↓, Cyclin D1↓, Bax↑, Bad↑, Bcl-2↓, Bclxl↓, cleaved Caspase-3↑, p-JAK2↓, p-STAT3↓	Lv et al., 2016
38	Kidney cancer	Salidroside	Mice with A498 xenografts model	40 and 80 mg/kg, i.p.	CDC25C↓, Cyclinn B1↓, Cyclin D1↓, Bax↑, Bad↑, Bcl-2↓, Bclxl↓, cleaved Caspase-3↑, p-JAK2↓, p-STAT3↓	Lv et al., 2016
39	Bladder cancer	Salidroside	UMUC-3	12.5, 25, and 50 μg/ml	p-AMPKα↑, p-ACC↑, p-mTOR↓, p-rpS6↓, cleaved LC-3II↑, p62↓	Liu et al., 2011
40	Bladder cancer	Ellagic acid	T24	33.7 μM	p-p38-MAPK↑, MEKK1↓, p-c-JUN↓, cleaved Capase-3↑	Qiu et al., 2013
41	Skin cancer	Gallic acid	A375.S2	250 μM	Caspase-3/8/9↑, ROS↑, Ca^2+^↑, AIF↑, Endo G↑, Fas↑, FasL↑, Bax/Bcl-2↑, Bax↑, Bcl-2↓	Lo et al., 2010
42	Cervical cancer	Gallic acid	HeLa	100 μM	Bax↑, Bax/Bcl-2↑, PARP↓, ROS↑, GSH↓	You et al., 2010
43	Brain cancer	Cordycepin	SH-SY5Y U-251	100, 200, and 300 μM	Caspase-3/9↑, Bax↑, p53↑, Bcl-2↓, Bax/Bcl-2↑, ROS↑,GPX↓, SOD↓, Catalase↓	Chaicharoenaudomrung et al., 2018
44	Gastric cancer	Gallic acid	AGS	2, 2.5, 3, and 3.5 μM	MMP-2/9↓, IκB↑, PI3K↓, Akt-1↓, p-Akt↓, Ras↓, Cdc42↓, rac1↓, RhoA↓, RhoB↑	Ho et al., 2010

↑: upgrade; ↓: downgrade.

### TTMs That Alter the Bcl-2/Bax Ratio

The Bcl-2 family has both proapoptotic and surviving members, which play important roles in regulating apoptosis (Rogers and Almenri, 2019). As an antiapoptotic protein, Bcl-2 is mainly distributed in the outer membrane of mitochondria, the inner surface of cell membrane, endoplasmic reticulum and nuclear membrane. On the contrary, Bax is a proapoptotic member of Bcl-2 family. Therefore, the proportional relationship between Bax/Bcl-2 plays a key role in mitochondrial mediated apoptosis (Li J. et al., 2018). In addition, many TTMs have been found to induce apoptosis by regulating the balance of Bcl-2 family members.

Cancer is recognized to be the result of three factors dysfunction (*loong*, *tripa* and *baekan*), especially under the inverse of the *loong*. According to the ancient Tibetan medicine classics, TTMs with tonic, clearing heat, and detoxifying functions are used to treat cancer. *O. sinensis* (**Figure 2**), which is known as Ya-er-zha-geng-bu (Tibetan: དབྱར་རྩྭ་དགུན་འབུ།), Dong-chong-xia-cao (Chinese name) or cordyceps (English name), is considered as one of the most valued Tibetan medicines (Qinghai Institute for Drug Control, 1996). In the theory of Traditional Chinese medicine and Tibetan medicine, *O. sinensis* is pungent flavor and warm-natured, and used for hundreds of years in traditional medicine as a tonic for the bronchitis, phthisis, pneumonia, lung heat, and impotence nocturnal emission (Dimaer, 2012). It’s worth noting that *O. sinensis* has a wide range of pharmacological properties, such as anti-inflammatory, cell cycle disruption, immune enhancement, induction apoptosis, *etc*. So it is widely used and concerned as an anticancer agent. Previous studies have found that water extract of *O. sinensis* combined with methotrexate could significantly prolong the survival time of mice inoculated with cancer cell sarcoma and inhibit the metastasis of tumor cells by inducing apoptosis (Nakamura, et al., 2003). An exopolysaccharide fraction from *O. sinensis* could significantly inhibit the metastasis of B16 melanoma cells and decreased the levels of Bcl-2 in the lungs and livers at a dose concentration of 120 mg/kg (Zhang et al., 2005). Treatment of A549 lung cancer cells with protein extract of *O. sinensis* could increase in Bax/Bcl-2 ratio, significantly upregulate mRNA levels of Bax, tumor necrosis factor-*α* (TNF-*α*), interleukin-1, and interleukin-12 (Wang Y.X. et al., 2018). *Tinospora cordifolia* (Willd.) Hook.f. & Thomson (Tibetan: Le-zhe) is one of the most widely used in TTM, has immunomodulatory, antitumor, anti-angiogenesis, and antimetastatic activity in various *in vivo* models. Aqueous ethanolic extract of *T. cordifolia* could block C6 glioma cells in G0/G1 phase and G2/M phase, inhibit the expression of G1/S phase specific protein cyclin D1 and antiapoptotic protein Bcl-xl, and thus produce its antiproliferation and apoptotic inducing effect in concentration range of 250–350 μg/ml (Ali and Dixit, 2013).

**Figure 3:**
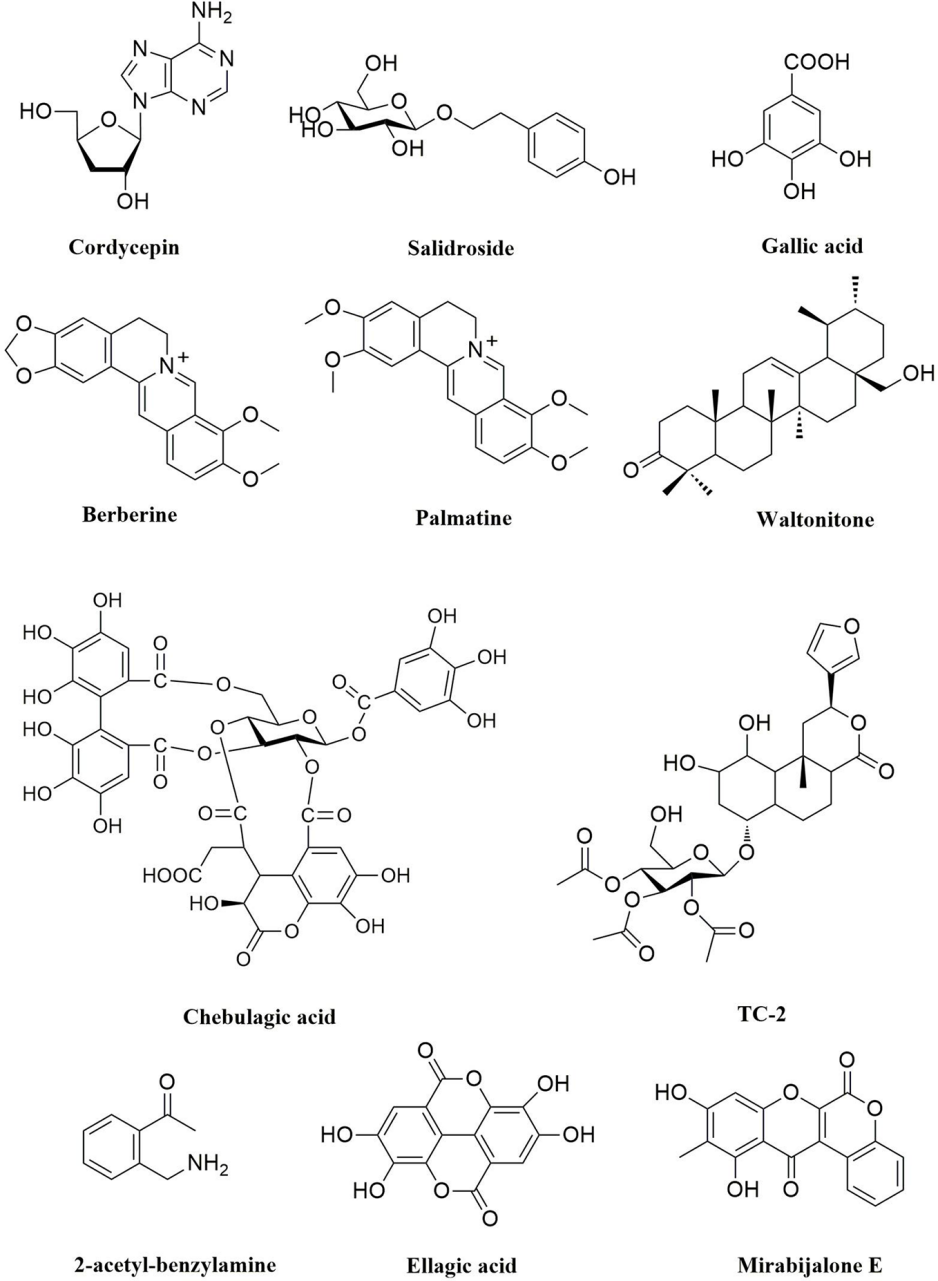
Natural compounds with anticancer activity in TTM by targeting apoptosis pathway.

Some active ingredients of TTMs that alter the Bcl-2/Bax ratio have been found and identified. Cordycepin, a 3-deoxyadenosine (**Figure 3**), is the predominant functional component of the fungus *Ophiocordyceps* species, has antitumor effects or apoptosis in brain cancer, human oral squamous cancer, thyroid carcinoma cancer, gallbladder cancer, liver cancer, breast cancer, and lung cancer (Wu et al., 2007; Chen Y. et al., 2010; Aramwit et al., 2015; Chaicharoenaudomrung et al., 2018). Cordycepin (5.11–15.34 μM) could inhibit cell proliferation and induce apoptosis in a dose-dependent manner. It was demonstrated that cordycepin could decrease the expression levels of Bcl-2 and caspase-3, increase the expression levels of proapoptotic protein Bax, and cleaved caspase-3 (Wang Z. et al., 2016). Notably, the study showed that cordycepin (125–500 μM) could induce the mitochondria mediated apoptosis signal pathway of human liver cancer HepG2 cells through upregulation of the ratio of Bax/Bcl-2, and initiating the FADD mediated signal pathway (Shao et al., 2016). Mirabijalone E (**Figure 3**), which was isolated from *M. himalaica*, has been reported to increase of Bax expression level and decrease of Bcl-2 level and activation of caspase-3 (Linghu et al., 2014). In addition, chebulagic acid (**Figure 3**) which was isolated from the fruits of *T. chebula*, could induce apoptosis by DNA fragmentation assay, PARP cleavage, cytochrome c release from the mitochondria and alteration of Bcl-2/Bax ratios in COLO-205 cell line (with an IC_50_ of 25μM) (Reddy et al., 2009).

### TTMs That Activate Caspases

Caspases are a family of cysteine proteases and play an important role in apoptotic and inflammatory signaling pathways. During the process of tumorigenesis, significant loss or inactivation of major members of the caspase family leading to impaired apoptosis induction, causing the serious imbalance of growth dynamics, and eventually to abnormal growth of human tumors (Rogers and Almenri, 2019). Caspases are divided into promoter groups (caspase-8/9/10) and executive groups (caspase-3/6/7). Re-activation of caspase to restore the apoptosis-induced pathway is a key molecular approach to the development of anticancer agents. Restoring apoptosis induction by caspase reactivation is a key molecular mechanism for the development of anticancer agents. Most studies have found that TTMs could induce apoptosis *via* caspase activation.

Saffron (**Figure 2**) is an edible spice and colorant found in the dried stigmas of *Crocus sativus* L. and has been used in TTM as an herb to treat various diseases, such as cancer. Over the past two decades, studies have been conducted on the therapeutic applications of saffron, which have been found to have anticancer, antitumor (*in vivo* and *in vitro*) and antimutagenic potential. Modern pharmacological studies have been proved that saffron can treat a variety of cancers, such as lung, breast, skin, and prostate cancers. In human lung cancer (A549 and H446), saffron extract (0.25–8.0 mg/ml) could suppress proliferation and induce apoptosis in a dose- and time-dependent manner and has significant anticancer effects *via* caspase-3/8/9 mediated cell apoptosis (Liu et al., 2014). The crocin family includes various glycosyl esters of which six types have been detected in saffron and is the main active substance of saffron. Previous studies have shown that crocin (0.2–1.0 mmol/L) could induce ovarian cancer HO-8910 cells’ apoptosis by increasing p53 and Fas/APO-1 expression and activating the apoptotic pathway regulated by Caspase-3 (Xia, 2015). In addition, crocin could induce apoptosis on human breast cancer cells (MCF-7) through a caspase-8-dependent mitochondrial pathway, involving p53 expression, Bax conformation, and mitochondrial membrane potential loss (Lu et al., 2015).

*Dracocephalum tanguticum* Maxim. (Labiatae) is a commonly used TTM for treating arthritis, hepatitis, and ulcer. In recent years, *D. tanguticum* has been used to treat glioblastomas. Wang et al. (2011) found that the chloroform extract of *D. tanguticum* stimulated caspase-3 cleavage and inhibited the expression of p21 protein with induction of glioblastomas cells (T98) apoptosis. The ethanol extract of *Meconopsis integrifolia* (Maxim.) Franch. and total flavonoid of *Oxytropis falcata* Bunge could block cell cycle processes and induce mitochondrial dependent apoptosis in human leukemia K562 cells and hepatoma SMMC-7721 cells by the release of cytochrome C, activation of Caspase-3/9 (Fan et al., 2015a; Chen et al., 2017). The ethanol extract of *Stellera chamaejasme* L. induced apoptosis significantly improved the activity of caspase-3/8/9, increased Fas and TNF-α expression (Liu X.N et al., 2012). *Carum carvi* L. essential oil has an efficient novel apoptosis inducer for human colon cancer cells (HT-29 and Huvec) by up-regulation Caspase-3 gene expression (Khatamian et al., 2019).

Ellagic acid (**Figure 3**), an important small molecular compound, was isolated from some TTMs, such as *P. emblica*, *T. chebula*, and *T. billerica*. Similarly, ellagic acid is a polyphenolic compound found in fruits and berries such as pomegranate, strawberry, raspberry, and blackberry. A large number of studies have reported the anticancer effects of ellagic acid on most types of cancer, such as colorectal, breast, prostate, lung, and liver cancers (Ceci et al., 2018). Hagiwara et al. (2010) found that ellagic acid activated apoptosis pathway associated with caspase-3 activation in human leukemia HL-60 cells. Notably, ellagic acid could enhance the chemotherapeutic sensitivity of 5-Fluorouracil and induce apoptosis by regulating the Bcl-2/Bax ratios and activating caspase-3 in colorectal carcinoma cells (HT-29) (Kao et al., 2012). In addition to the mechanisms mentioned above, ellagic acid induced apoptosis by regulating ROS, PI3K/Akt, JAK2/STAT3, MAPK, and NF-κB pathway in cancer cells (Bisen et al., 2012; Ceci et al., 2018).

### TTMs That Activate Reactive Oxygen Species

Reactive oxygen species (ROS) are substances produced by all aerobic cells to regulate cell development, growth, survival and death. ROS are generally present in all aerobic cells in relative balance with biochemical antioxidants. When this balance is disrupted by mitochondria excess production of ROS and/or depletion of antioxidants, oxidative stress may occur, which eventually leads to mitochondrial swelling, depolarization of mitochondrial membrane potential, and release of apoptosis-inducing proteins (Wang R. et al., 2016; Chaicharoenaudomrung et al., 2018). Oxidative stress is a major apoptotic stimulus for cancer cells, which require particularly high energy metabolism in the process of rapid growth and proliferation. Therefore, the production of ROS may enhance the proapoptotic mechanism of cancer cells and provide important targets for the treatment of cancer. TTMs have been reported to induce apoptosis of cancer cells by production of ROS.

*P. emblica*, a euphorbiaceous plant, is widely distributed in subtropical and tropical regions of China, India, Indonesia, and Malay Peninsula (Liu X.L. et al., 2012). The dried fruits of *P. emblica* is one of the famous plants used in traditional medicinal systems such as Ayurvedic medicine, Tibetan traditional medicine, Chinese herbal medicine, and Thai traditional medicine (Zhang Y.J. et al., 2004; Ngamkitidechakul et al., 2010). In traditional medicine Tibetan system, *P. emblica* is called “Ju-ru-re” (Tibetan: སྐྱུ་རུ་ར།). It is the most frequently used formulations in TTM (Li et al., 2018a). The extensive use of *P. emblica* in traditional medicines and food products has led to a large number of pharmacological activity studies. Up to now, a large number of biological activities have been reported, such as anti-inflammatory, antioxidant, antitumor, and immunomodulatory effects. It is noted that the aqueous extract of *P. emblica* (25–100 μg/ml) could induce apoptosis on human hepatoma cells (HepG2) by reducing production of ROS and increasing the levels of glutathione (Shivananjappa and Josi, 2012).

*Swertia mussotii* Franch., which is known as “Di-da” (Tibetan: ཏིག་ཏ།), was reported in the classic book of Tibetan medicine “Jing Zhu Materia Medica” that *S. mussotii* has the clearing heat and detoxifying functions. Recent studies have shown that *S. mussotii* has significant anticancer activity. Wang H. et al. (2018) reported that ethanol extract of *S. mussotii* was able to induce apoptosis in gastric cancer cells (MGC-803 and BGC-823) through depolymerization of cytoskeletal filaments, S phase arrest, disrupted mitochondrial transmembrane potential and increased cytoplasmic levels of ROS. Similarly, *Meconopsis horridula* Hook. f. & Thomson ethanol extract induced murine leukemia L1210 cell apoptosis and inhibited proliferation through G2/M phase arrest, and ROS were involved in the process (Fan et al., 2015b). Gallic acid, 3,4,5-trihydroxybenzoic acid (**Figure 3**), which can be found in various natural products, such as green tea, grapes, *Punica granatum* L., *P. emblica*, *Galla chinensis* Mill., and many other fruits plants. Gallic acid known to affect several pharmacological and biochemical pathways have strong antioxidant, antimutagenic, anti-inflammatory, and anticancer properties (Karimi-Khouzani et al., 2017; Limpisophon and Schleining, 2017; Silva et al., 2017; Ahmed et al., 2018). Therefore, gallic acid has been recognized as an inducer of apoptosis in cancer cell lines. It has been reported that gallic acid could induce apoptosis by ROS-dependent mitochondrial pathway in most cancer cells, such as colon cancer HCT-15 cells, small cell lung cancer H446 cells, prostate cancer DU145 cells, cervical cancer HeLa cells, melanoma A375.S2 cells (Lo et al., 2010; You et al., 2010; Subramanian et al., 2016; Wang R. et al., 2016). TC-2 (**Figure 3**) is a new clerodane diterpenoid from *Tinospora cordifolia* (Willd.) Hook.f. & Thomson. It has been confirmed that TC-2 induced apoptosis of colon cancer cells (HCT) cells by triggering ROS production (Sharma et al., 2018). In addition, cordycepin (**Figure 3**) inhibited cell growth and induced apoptosis on human brain cancer cells (SH-SY5Y and U251), related to ROS-mediated apoptosis pathway, accompanied by upregulation the expression of P53, Bax, Caspase-3/9, and downregulation the levels of Bcl-2, GPX and SOD (Chaicharoenaudomrung et al., 2018).

### TTMs Targeting PI3K/Akt and JAK2/STAT3

The phosphatidylinositol-3 kinase (PI3K) signaling pathway is involved in many cancer processes. Meanwhile, the serine/threonine specific protein kinase Akt, the main downstream effector of PI3K, is frequently activated (Chen, 2016). In addition, Akt is a key regulator of the survival, proliferation, differentiation, apoptosis, and metabolism of cancer cells. Therefore, in recent years, PIK/Akt has received considerable attention in cancer research. Signal transducer and activator of the activator of transcription 3 (STAT3) can regulate the cancer cell proliferation, apoptosis and survival by activating Janus kinase 2 (JAK-2) (Lv et al., 2016; Hoshyar and Mollaei, 2017).

*Rhodiola* species are genera of perennial plants of the family Crassulaceae, which grow in high-altitude and cold areas in China, such as Tibet, Sichuan, Yunnan, and Qinghai (Xia et al., 2005). Among these species, *Rhodiola crenulata* and *R. kirilowii* are the most commonly used species of Hong-jing-tian as folk medicine in China. Modern studies have shown that *Rhodiola* species possesses a wide range of pharmacological activities, such as anti-altitude sickness, immunomodulatory, anti-inflammatory, antifatigue, and anticancer activities (Kumar et al., 2010; Tao et al., 2019). It is noteworthy that *R. kirilowii* was reported to show potential anticancer activity. It has been found that ethanol extract of *R. kirilowii* in the concentration range of 10–40 mg/ml inhibited human breast cancer cells (MDA-MB-231 and HUVEC) migration and invasion, and significantly decreased phosphorylation of Akt and PKC on PI3K/Akt signaling pathway (Wang and Lin 2015). Salidroside (**Figure 3**), a p-hydroxyphenethyl-*β*-D-glucoside, was isolated from *Rhodiola* species, has been reported to exhibit extensive anticancer effects. It has been verified that salidroside induced apoptosis and autophagy in human colorectal cancer cells (HT-29) through inhibition of PI3K/Akt/mTOR pathway at 0.5, 1 and 2 mM (Fan et al., 2016). In addition, salidroside also induced apoptosis in renal cell carcinoma (A498 and 786-0), and reduced the levels of p-STAT3 and p-JAK2 at a concentration of 60 μM (Lv et al., 2016).

*Pterocephali herba* is the whole herb of the perennial plant *Pterocephalus hookeri* (C.B. Clarke) Höeck, a member of the Dipsacaceae family. *P. hookeri* has clearing heat and detoxifying functions in TTM. It is mainly used to treat rheumatoid arthritis and influenza. Recent research found that n-butanol extracts of *P. hookeri* with 50–200 μg/ml inhibited proliferation and induced apoptosis on Hep3B cancer cells, blocked PI3K/Akt pathway, and regulated the levels of Bcl-2 family proteins (Guo C. et al., 2015). Waltonitone (**Figure 3**), a pentacyclic triterpenoid of ursane type compound, was isolated from *Gentiana waltonii*, inhibited the cell growth, and induced apoptosis on hepatocellular carcinoma a BEL-7420 cells by modulating Akt and ERK_1/2_ pathway (Zhang et al., 2009). In addition, 2-acetyl-benzylamine (**Figure 3**) isolated from *Justicia adhatoda* L. (syn. *Adhatoda vasica* Nees) (0.42, 0.84, and 1.68 mM) could induce apoptosis, inhibit the expression of JAK2/STAT3, and regulate Bcl-2/Bax ratios in MOLM-14 and NB-4 cells (Balachandran, et al., 2017).

### TTMs That Downregulate the NF-*κ*B Pathway

The nuclear factor-kappa B (NF-*κ*B) pathway is one of the most important cellular signal transduction pathways involved in immunity, inflammation, proliferation, and apoptosis. Most of studies showed that NF-*κ*B played a key role in cancer progression. Activation of NF-*κ*B leads to either upregulation of antiapoptotic genes (FLIP, cIAP, survivin, Bcl-2, and Bcl-XL) or downregulation of apoptotic genes (Li et al., 2017). Therefore, the combination of chemotherapy drugs with NF-*κ*B inhibitors is considered to be an effective therapeutic strategy for the treatment of cancer.

*Berberis aristata*, known as Ji-er-wa (Tibetan: སྐྱེར་པའི་བར་ཤུན།) in TTM, has been widely used to treat inflammation and diabetes (Belwal et al., 2020) due to its anti-inflammatory and immune-potentiating properties. Serasanambati et al. (2015) found that different concentrations (125, 250, and 500 μg/ml) of the methanolic extracts of *B. aristata* could significantly inhibit cell migration and induce apoptosis in human breast cancer cells (MCF-7). Berberine and palmatine are isoquinoline alkaloids (**Figure 3**), which can be extracted from some medicinal plants, such as *Berberis aristata*, *B. kansuensis*, *B. diaphana*, *B. vernae*, and *Coptis chinensis* (Serasanambati et al., 2015; Li et al., 2018b; Neag et al., 2018; Sheng et al., 2018). Berberine exhibits multiple biologic effects with low toxicity, and the antitumor activities in various human cancer cells have been reported (Yip and Ho, 2013; Zhao et al., 2017; Yao et al., 2018). Berberine (80–160 µmol/l) induced apoptosis by suppressing NF-*κ*B nuclear translocation *via* Set9-mediated lysine methylation, decreasing the levels of miR21 and Bcl-2 (Hu et al., 2012). Meanwhile, berberine (10, 50, and 100 μM) could inhibit the growth of HepG2 cells by promoting apoptosis through the NF-*κ*B p65 pathway (Li et al., 2017). It is worth noting that palmatine-induced apoptosis was associated with decreased activation of NF-*κ*B and downstream target gene FLIP (Hambright et al., 2015).

*Gentianopsis paludosa* (Hook.f.) Ma is an annual Gentianaceae plant. As a traditional Tibetan medicinal material, it has been widely used as an herb in China because of its clearing heat and detoxifying functions. Lu et al. (2016) found that ethanol extract of *G. paludosa* could induce apoptosis of colon cancer cells (SW480), and the mechanism might be partly related to the NF-*κ*B signaling pathway. In addition, some compounds of TTMs can also downregulate the NF-*κ*B pathway. Cordycepin (75, 110, and 145 μmol/L) could inhibit the proliferation and induct the apoptosis of A549 cells dose-dependently, increase the expression of Bax and cleaved caspase-3, decrease the expression of Bcl-2, and the mechanism of action was achieved by inhibiting the NF-*κ*B pathway (Zhang et al., 2015). Choi et al. (2009) showed that gallic acid (5, 10, 25, and 50 μM) inhibited inflammatory responses caused in A549 lung cancer cells by other stimuli, including lipopolysaccharide, IFN-*γ*, and interleukin-1*β*, and further downregulated the expression of NF-*κ*B-regulated antiapoptotic genes.

### TTMs That Mediate the MAPK Pathway

Mitogen-activated protein kinases (MAPKs) belong to an evolutionarily conserved and ubiquitous signal transduction superfamily of Ser/Thr protein kinases. The MAPK pathway is involved in the growth, development, proliferation, and differentiation of various cells. The MAPK pathway, involving its major subgroups ERK1/2, JNK, and p38 MAPK, is involved in physiological processes such as the growth, development, proliferation, and differentiation of various cells (Zhao H. J. et al., 2015). More and more studies have shown that the MAPK pathway plays important roles in the process of apoptosis transduction and is significantly related to the occurrence and development of breast, ovarian, esophageal, colon, stomach, and liver cancers (Yoon et al., 2018).

It is worth noting that *Phyllanthus emblica* L. can induce cancer cell apoptosis through the MAPK pathway. Zhao H.J. et al. (2015) found that the tannin fraction of *P. emblica* (15, 30, and 60 mg/L) dose-dependently induced apoptosis of human lung squamous carcinoma cells (NCI-H1703) by suppressing the expression of p-ERK1/2, MMP-2/9, upregulating the expression of p-JNK. Therefore, the tannin fraction of *P. emblica* induced apoptosis *via* the MAPK/MMP pathways. Furthermore, berberine, a famous small molecule compound from TTM, also has the function of regulating MAPK pathway. Zheng et al. (2014) and Kim et al. (2015) reported similar results that berberine-induced apoptosis was mediated by activation of the p38 MAPK signaling pathway *via* the death receptor ligand FOXO3a, p53, and FasL. In another study, berberine also promoted the rate of apoptosis of NSCLC cells by the suppression of the MMP-2, Bcl-2/Bax, and modulating the miR-19a/TF/MAPK signaling pathway (Chen et al., 2019).

### TTMs That Activate AMPK Pathway

The AMP-activated protein kinase (AMPK), which is a conserved heterotrimeric protein kinase, is an important “energy sensor” regulating intracellular metabolism and energy balance and is very sensitive to changes in AMP/ATP ratio (Pan et al., 2017). AMPK is rapidly activated when cellular energy metabolism is abnormal, such as starvation, hypoxia, and ischemia (Ortiz et al., 2014). A series of studies have found that AMPK has strong proapoptotic potential under activated conditions. In summary, AMPK can be an important target for the treatment of cancer.

In addition to the mechanisms described above, berberine can also be used to treat cancer by activating AMPK. It was found that after berberine (12.5 and 50 μM) pretreatment of hepatocellular carcinoma cells (HepG2), the levels of p-AMPK and p-Akt were significantly increased. In addition, activation of AMPK was associated with caspase-dependent mitochondrial pathway apoptosis, coupled with mitochondrial cytochrome c release and activation of Caspase-3/9, with a dose-dependent increase in the Bax/Bcl-2 ratio. Therefore, berberine could selectively inhibit HepG2 cells’ growth by inducing AMPK-mediated caspase-dependent mitochondrial pathway apoptosis (Yang and Huang, 2013). Moreover, berberine (10 and 80 μM) enhanced Doxorubicin sensitivity of drug-resistant in MCF-7/MDR breast cancer cells *via* AMPK/HIF-1*α*/P-gp pathway and directly induced apoptosis through the AMPK/p53 pathway (Pan et al., 2017).

Salidroside has a wide range of pharmacological activities, especially antiplateau hypoxia and immune-enhancing effects. It has been reported that salidroside can reduce superoxide dismutase (SOD) level in the mitochondria and improve endurance exercise performance. Therefore, it can be considered that salidroside reduces the production of SOD due to its effect on oxygen consumption, resulting in the change of ATP and finally the activation of AMPK. This was discovered in bladder cancer cells (UMUC3) by Liu et al. (2011). It is worth noting that salidroside could induce the autophagy-related apoptosis on human acute monocytic leukemia cells (THP-1 and U937) through AMPK activation *via* downregulating p62, p-PI3K, p-AKT, and p-mTOR expressions and upregulating Beclin1, LC3II and AMPK expressions (Ge et al., 2019).

## Conclusion Remarks

Traditional medicines are the gifts of nature to humans. Many drugs that are commonly used in modern medicine, such as artemisinin, paclitaxel, camptothecin, and ephedrine, are derived directly or indirectly from these natural medicines. TTM is an ancient health system and part of the world’s traditional medical system. This system uses various treatments and personalized approaches to prevent and treat a wide range of diseases, especially chronic diseases, such as cancer.

In this review, we attempt to summarize the traditional Tibetan medical theory on the knowledge and treatment of cancer. The results showed that, in TTM, the direct cause of cancer is the shrinking and aggregating of “bad blood” owing to the reverse effect of *loong* (**Figure 1**). In addition, we review the natural Tibetan medicines traditionally used in the Tibetan system of medicine for cancer treatment. More importantly, some TTMs and their effects on apoptotic pathways are summarized in **Table 4**. Most TTMs exert anticancer effects through multiple components and multiple pathways. As previously mentioned, apoptosis is one of the main mechanisms by which TTM induces cancer cell death. Therefore, the molecular mechanisms of Tibetan medicine targeting apoptosis pathways are worthy of further study. However, in addition to apoptosis pathway targets, other cell death pathways may be triggered by TTMs. For example, some TTMs show anticancer activity by enhancing immunity. In order to fully evaluate the anticancer potential of these TTMs and their active ingredients, multidisciplinary approaches should be integrated to conduct pharmacological studies and reveal their mechanisms of action.

**Table 4:** Summary of traditional Tibetan medicine and apoptotic pathway targets.

Apoptotic pathway targets	Traditional Tibetan medicine
Bcl-2/Bax ratio	Protein extract of *Cordyceps sinensis* (Wang Y.X. et al., 2018), chloroform extract of *Dracocephalum tanguticum* (Wang et al., 2011), aqueous ethanolic extract of *Tinospora cordifolia* (Mishra and Kaur, 2013), *Stellera chamaejasme* with liquor (Ma et al., 2013), n-butanol extract of *Pterocephalus hookeri* (Guo C.X. et al., 2015), mirabijalone E (Linghu et al., 2014), crocin (Hoshyar and Mollaei, 2017), salidroside (Fan et al., 2016), gallic acid (Verma et al., 2013), berberine (Chen et al., 2019), 2-acetyl-benzylamine (Balachandran et al., 2017), chebulagic acid (Reddy et al., 2009), and ellagic acid (Ceci et al., 2018)
Caspases	Chloroform extract of *Dracocephalum tanguticum* (Wang et al., 2011), total flavonoid of *Oxytropis falcata* (Chen et al., 2017), ethanol extract of *Meconopsis integrifolia* (Fan et al., 2015a), ethanol extract of *Stellera chamaejasme* (Liu X.N. et al., 2012), essential oil of *Carum carvi* (Khatamian et al., 2019), dichloromethane extract of *Carthamus tinctorius* (Arpornsuwan et al., 2012), crocin (Hoshyar and Mollaei, 2017), cordycepin (Yoon et al., 2018), salidroside (Hu et al., 2010), gallic acid (VerMa et al., 2013), berberine (Yao et al., 2018), mirabijalone E (Linghu et al., 2014), 2-acetyl-benzylamine (Balachandran et al., 2017), and ellagic acid (Ceci et al., 2018)
ROS	Ethanol extract of *Swertia mussotii* (Wang H. et al., 2018), ethanol extract of *Meconopsis horridula* (Fan et al., 2015b), ethanol extract of *Meconopsis integrifolia* (Fan et al., 2015a), cordycepin (Chaicharoenaudomrung et al., 2018), salidroside (Zhao G. et al., 2015), gallic acid (Wang R. et al., 2016), berberine (Xie et al., 2015), TC-2 (Sharma et al., 2018), and ellagic acid (Ceci et al., 2018)
PI3K/Akt	Ethanol extract of *Rhodiola kirilowii* (Wang and Lin, 2015), cordycepin (Yoon et al., 2018), gallic acid (VerMa et al., 2013), berberine (Chen, 2016), salidroside (Ge et al., 2019), waltonitone (Zhang et al., 2009), and ellagic acid (Ceci et al., 2018)
JAK2/STAT3	Salidroside (Kang et al., 2018), 2-acetyl-benzylamine (Balachandran et al., 2017), crocin (Hoshyar and Mollaei, 2017), berberine (Li J. et al., 2018), and ellagic acid (Ceci et al., 2018)
NF-κB	Ethanol extract of *Gentianopsis paludosa* (Lu et al., 2016), polysaccharide of *Cordyceps sinensis* (Jayakumar et al., 2014), cordycepin (Yoon et al., 2018), salidroside (Li and Chen, 2017), gallic acid (Verma et al., 2013), berberine (Li J. et al., 2018), palmatine (Hambright et al., 2015), and ellagic acid (Ceci et al., 2018)
MAPK	Tannin fraction of *Phyllanthus emblica* (Zhao H.J. et al., 2015), polysaccharide of *Cordyceps sinensis* (Jayakumar et al., 2014), cordycepin (Yoon et al., 2018), salidroside (Zhao G. et al., 2015), berberine (Chen et al., 2019), waltonitone (Zhang F.G. et al., 2010), and ellagic acid (Ceci et al., 2018)
AMPK	Salidroside (Ge et al., 2019) and berberine (Pan et al., 2017)

In addition, current research on TTMs is insufficient and limited. First, according to statistics (Jia and Zhang, 2016), 3,105 natural medicines have been used in the Tibetan medicine theory system. However, only 40 species have been demonstrated to possess cancer-related biological activity, and most species still lack sufficient experimental evidence. For example, brag-zhun is a natural exudate from rock stratum, which sometimes contains animal feces. Brag-zhun and its preparations are commonly used in Tibetan medicines for cancer therapy. However, to date, reports on the biological activity of the medicine associated with cancer are unavailable. Similarly, *Swertia chirayita* (Roxb. ex Fleming) H. Karst. and *Halenia elliptica* D.Don also lack cancer-related research. Given the high frequency of natural medicines being used in the treatment of cancer, supplementing these gaps in research is necessary. Secondly, although some compounds that are isolated from TTMs exhibit cancer-related biological activities, their cellular and molecular mechanisms, and possible synergies among these compounds have not been clearly elucidated. Third, TTM mainly uses prescriptions to treat cancer in clinic, but relevant research to support their application is limited. Only studies involving *Yukyung Karne* have been reported. Addressing these limitations in future research is necessary. Moreover, although some Tibetan herbal medicines can induce the death of cancer cells through the apoptotic pathways *in vitro*, these herbs have a weak anticancer effect on animal models. Therefore, *in vivo* experiments are necessary to verify the anticancer effects and molecular mechanisms of these TTMs.

In conclusion, this review provides the first compilation of data on TTM for cancer treatment. We found that some TTMs (*e.g.*, *O. sinensis*, *P. emblica*, and *Rhodiola kirilowii*) and their active ingredients (*e.g.*, cordycepin, salidroside, and gallic acid) have good anticancer activity. The molecular mechanisms are mainly through targeting some apoptotic pathways in cancer, for example, Bcl-2/Bax, caspases, PI3K/Akt, JAK2/STAT3, MAPK, and AMPK. These herbs and natural compounds would be potential drug candidates for cancer treatment and deserve further research and development.

## Author Contributions

CT and C-CZ: collected and organized the data and wrote the paper. HY and X-YW: collected the data. Z-JG: wrote the Tibetan names of natural medicines. YZ: amended the paper. YL and GF: conceived and designed the study and amended the paper.

## Conflict of Interest

The authors declare that the research was conducted in the absence of any commercial or financial relationships that could be construed as a potential conflict of interest.
